# Conventional versus Analgesia-Oriented Combination Sedation on Recovery Profiles and Satisfaction after ERCP: A Randomized Trial

**DOI:** 10.1371/journal.pone.0138422

**Published:** 2015-09-24

**Authors:** Seokyung Shin, Tak Geun Oh, Moon Jae Chung, Jeong Youp Park, Seung Woo Park, Jae Bok Chung, Si Young Song, Jooyoun Cho, Sang-Hun Park, Young Chul Yoo, Seungmin Bang

**Affiliations:** 1 Department of Anesthesiology and Pain Medicine, Severance Hospital, Anesthesia and Pain Research Institute, Yonsei University College of Medicine, 50–1 Yonsei-ro, Seodaemun-gu, 03722, Seoul, Korea; 2 Division of Gastroenterology, Department of Internal Medicine, Severance Hospital, Institute of Gastroenterology, Yonsei University College of Medicine, 50–1 Yonsei-ro, Seodaemun-gu, 03722, Seoul, Korea; Universita' degli Studi di Napoli Federico II, ITALY

## Abstract

**Background:**

The importance of providing effective analgesia during sedation for complex endoscopic procedures has been widely recognized. However, repeated administration of opioids in order to achieve sufficient analgesia may carry the risk of delayed recovery after propofol based sedation. This study was done to compare recovery profiles and the satisfaction of the endoscopists and patients between conventional balanced propofol sedation and analgesia-oriented combination sedation for patients undergoing endoscopic retrograde cholangiopancreatography (ERCP).

**Methods:**

Two hundred and two adult patients scheduled for ERCP were sedated by either the Conventional (initial bolus of meperidine with propofol infusion) or Combination (repeated bolus doses of fentanyl with propofol infusion) method. Recovery profiles, satisfaction levels of the endoscopists and patients, drug requirements and complications were compared between groups.

**Results:**

Patients of the Combination Group required significantly less propofol compared to the Conventional Group (135.0 ± 68.8 mg vs. 165.3 ± 81.7 mg, P = 0.005). Modified Aldrete scores were not different between groups throughout the recovery period, and recovery times were also comparable between groups. Satisfaction scores were not different between the two groups in both the endoscopists and patients (P = 0.868 and 0.890, respectively).

**Conclusions:**

Considering the significant reduction in propofol dose, the non-inferiority of recovery profiles and satisfaction scores of the endoscopists and patients, analgesia oriented combination sedation may be a more safe yet effective sedative method compared to conventional balanced propofol sedation during ERCP.

## Introduction

Sedation for gastrointestinal endoscopic procedures has become commonplace during the past decade, and many attempts have been made to find a safe and yet effective combination of sedatives and analgesics. Despite considerable dispute, propofol has gained overall popularity as the sedative of choice by many endoscopists and anaesthesiologists, and has largely replaced the traditional use of benzodiazepines.[1–3] However, its lack of analgesic property, narrow therapeutic window and absence of a reversal agent have been recognized as the cause of adverse events related to oversedation.[4]

In an attempt to reduce these complications, balanced propofol sedation (BPS) was proposed as a method that would provide safe and effective sedation by combining a low-dose of propofol with an opioid analgesic and/or benzodiazepine.[5] While the concept of BPS has been generally well received by the medical society, the issue of which and how much opioid analgesic should be combined with propofol is still under debate.[6,7] The conventionally used opioid was meperidine, and many clinicians administered this agent routinely for pain control during endoscopic procedures. However, meperidine is quickly losing its place as an analgesic for interventional procedures due to its many side effects and low efficacy.[8,9] The toxic potential of meperidine renders it inappropriate for use during endoscopic retrograde cholangiopancreatography (ERCP), as such procedures are usually accompanied by severe pain and discomfort which may require repeated analgesics administration.[10] On the other hand, we found in a recent retrospective study that respiratory events were significantly reduced when effective analgesia was provided during ERCP by giving bolus doses of fentanyl as needed.[11] However, repeated doses of fentanyl may carry the risk of prolonged recovery time, which could be a problem in terms of patient safety and efficiency.

This randomized, controlled trial was conducted to compare recovery profiles between conventional BPS using a single dose of meperidine and a combination method using repeated doses of fentanyl during propofol-based sedation for ERCP. We hypothesized that a combination method would be able to offer satisfactory sedation without prolonging recovery times compared to conventional BPS. Satisfaction levels of the endoscopists and patients as well as drug requirements, cardiovascular and respiratory events were also assessed and compared between the two groups.

## Methods

### Ethics statement

The study protocol was approved by the Institutional Review Board and Hospital Research Ethics Committee of Severance Hospital, and registered at http://clinicaltrials.gov (registration number NCT01840371).

### Study population

Adult patients of American Society of Anesthesiologists (ASA) physical status I ~ IV that were scheduled for elective ERCP between April 2013 and October 2013 were recruited for this study. All patients provided written informed consent. Pregnant or breastfeeding patients and those with known allergies to eggs, soy beans or sulfites were excluded from this study. Patients that had received sedation for another procedure within 24 hours prior to ERCP, were unable to provide informed consent or were under the age of 20 years were also excluded.

### Study design and sedation protocols

The targeted level of sedation was MOAA/S (Modified Observer’s Assessment of Alertness/Sedation Scale) score 3 to 4 in all patients. Sedation in the Conventional Group was done by administrating 25 mg of IV meperidine (Pethidine, Jeil Pharmaceutical Co. Lt., Daegu, Korea) and 1 mg kg^-1^ of propofol (Pofol, Dong Kook Pharmaceutical Co. Ltd., Seoul, Korea) initially, followed by propofol infusion at a maintenance rate of 60 μg min^-1^kg^-1^ using an automated pump (Terufusion® Syringe Pump TE-331, Terumo Corporation, Tokyo, Japan). Additional doses of 10 mg of propofol were given when patients complained of discomfort/pain, or when the endoscopist demanded deeper sedation. Patients of the Combination Group were given 1 μg kg^-1^ of IV fentanyl (Hana pharmaceutical, Korea) and 0.4 mg kg^-1^ of propofol initially, followed by propofol infusion at a maintenance rate of 30 μg min^-1^ kg^-1^. Additional doses of 10 mg of propofol were given during insufficient sedation, while bolus doses of 0.5 μg kg^-1^ of fentanyl were given when additional analgesia was needed. The need for additional sedation and analgesia was judged by the attending anesthesiologist based on the visual observation of patient irritability or excessive movement. Benzodiazepines were not used in our study.

Enrolled patients were randomly assigned to either the Conventional Group (n = 100) or the Combination Group (n = 102) by means of a table of random numbers generated by 4 block design with the SAS program (SAS institute Inc., Cary, NC, USA). The allocation sequence was assigned to each patient in sealed envelopes with equal randomization (1:1 for the two groups) at the point of sedation induction. The patients, all study investigators and medical personnel except the attending anesthetist providing sedation were blinded to group assignment. All ERCP procedures were performed by 5 endoscopists of similar experience (who performed more than 300 ERCPs annually for more than 3 years) and sedation was done by a qualified anesthesiology staff member trained for procedural sedation outside the operating theater that was not involved in the study.

### Patient monitoring

All endoscopic procedures were performed in a room dedicated to ERCP, fully equipped for advanced cardiac life support. Patients arrived at the endoscopy room with secure IV access, and nasal oxygen was supplied at 3 L min^-1^. Patient monitoring included non-invasive blood pressure measurement every 5 minutes, pulse oximetry (SpO_2_) and electrocardiography. Recovery score assessment using the modified Aldrete score[12] was initiated immediately after the procedure ended (0 minutes), and was followed at 5, 15 and 25 minutes post-procedure by a nurse that was blinded to the sedation regimen at the endoscopy recovery unit.

### Outcome measurements and definitions

The primary outcome of this study was to compare recovery profiles between the two groups. In addition to assessing recovery based on the modified Aldrete score at the aforementioned time points, time required for full recovery was noted. Recovery time was defined as the time interval between scope withdrawal and the modified Aldrete score reaching 10 points.

The endoscopists gave a satisfaction score at the end of the procedure, and patients were asked by a nurse that was blinded to the sedation regimen to give a score upon full recovery at the endoscopy recovery unit. Satisfaction scores were initially given on a verbally administered numerical rating scale of 0 to 100.

Procedure duration was defined as the time between scope insertion and scope withdrawal. Adverse respiratory events such as desaturation and apnea were also recorded by the attending anesthetist and compared between the two groups. Desaturation was defined as SpO_2_ lower than 90% for more than 10 seconds that required either an increase in O_2_ supply, chin lift, jaw thrust maneuver or assisted mask ventilation at the discretion of the attending anesthetist. Apnea was defined as the cessation of voluntary respiration for more than 30 seconds under visual observation. Hypertension due to severe pain or undersedation was defined as more than a 20% increase of mean blood pressure (MBP) from baseline, and hypotension was defined as systolic blood pressure (SBP) < 90 mmHg or MBP < 60 mmHg. Bradycardia and tachycardia were each defined as heart rate (HR) < 60 bpm and > 100 bpm, respectively.

Post-procedure pain scores were recorded on a visual analogue scale (VAS) of 0–10 at 6, 12, 18 and 24 hours. The number of times rescue analgesics or antiemetics were administrated were recorded during intervals of 0–12 hours and 12–24 hours after the procedure.

### Statistical analysis

A Sample size was calculated based on the recovery times after propofol monosedation reported in a previous study.[7] To detect a 20% difference in recovery time with a non-inferiority design between groups, 100 patients per group would be required to obtain a power of 85%, assuming a type I error of 0.05. Continuous variables with normal distribution were analyzed with the independent two sample t-test. Continuous variables that were not normally distributed were analyzed with the Mann-Whitney U Test and expressed as median and interquartile range (IQR). Categorical variables were analyzed by the Chi-square or Fisher’s exact test. All statistical analyses were performed with IBM SPSS Statistics 20.0 (IBM Corp., Armonk, NY, USA). A P value of < 0.05 was considered statistically significant. Data are described as mean ± SD or number of subjects.

## Results

**Fig 1:**
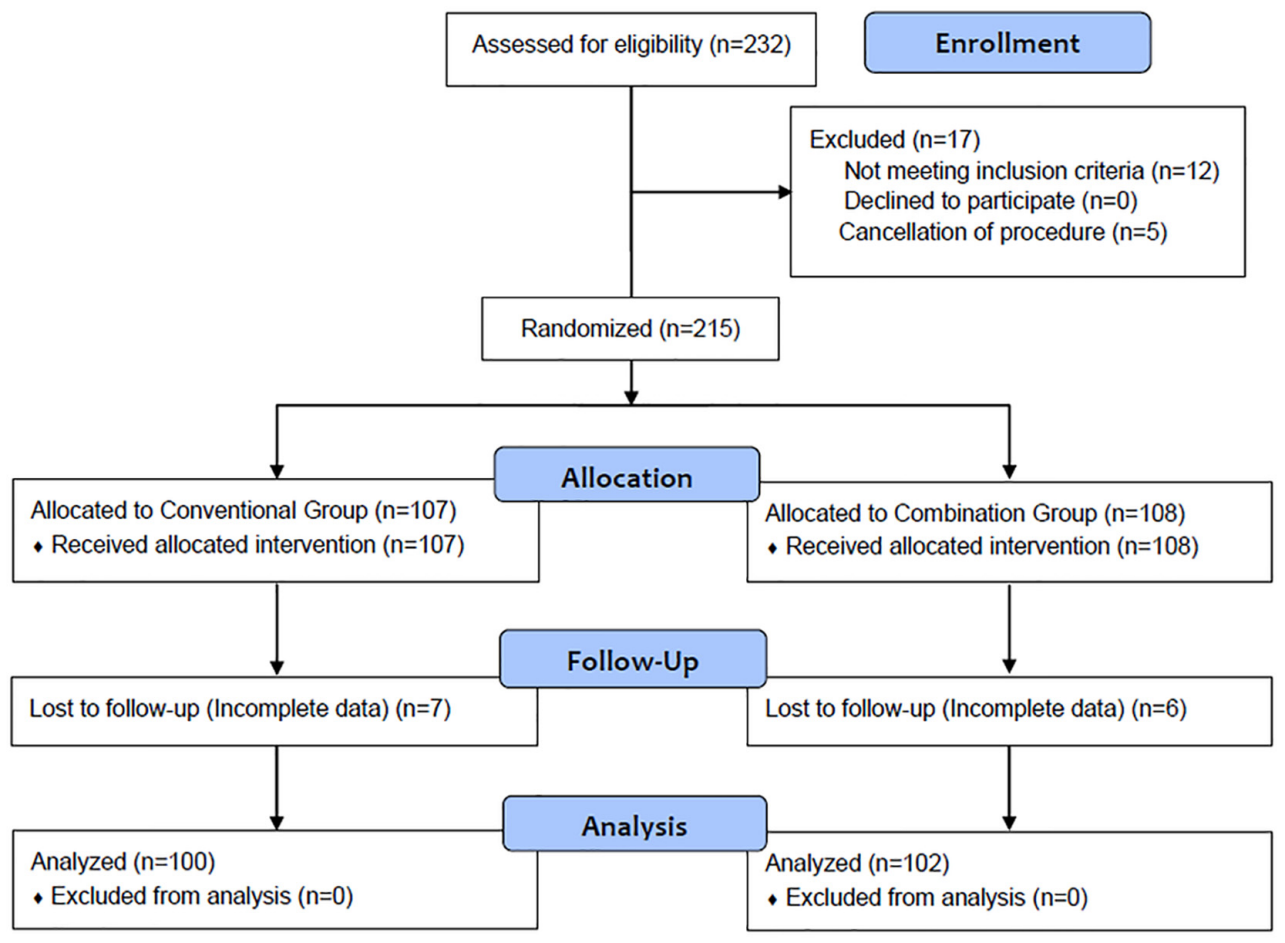
CONSORT flowchart.

The CONSORT flow diagram of this study is shown in Fig 1. Of the 232 patients that were assessed for eligibility, 12 patients that received propofol sedation for endoscopic ultrasound just before ERCP and another 5 patients in whom ERCP was cancelled were excluded from this study. The remaining 215 patients were randomly assigned to either the Conventional Group (n = 107) or the Combination Group (n = 108). After randomization, 7 patients in the Conventional Group and 6 patients in the Combination Group were dropped from analysis due to incomplete medical records or missing data at the time of analysis. The remaining 202 patients (100 and 102 patients of the Conventional Group and Combination Group, respectively) successfully completed the study and were included for analysis.

### Patient characteristics

Patient characteristics and procedure-related data are listed in Table 1. While there were no difference in patient characteristics with regard to gender, body mass index, history of smoking, alcohol intake or snoring and ASA classification, the patients in the Combination Group were significantly older than those of the Conventional Group (P = 0.025). Reason for ERCP and type of performed ERCP were not significantly different between the two groups.

**Table 1:** Patient characteristics. Values are mean ± SD or number (%) of patients. ERCP, endoscopic retrograde cholangiopancreatography. ASA, American Society of Anesthesiologists

Variables	Conventional Group (n = 100)	Combination Group (n = 102)	P-value
Age (years)	60.9 ± 14.6	65.3 ± 13.8	0.025^•^
Gender (male/female)	52 / 48	60 / 42	0.329^••^
Body mass index (kg/m^2^)	22.8 ± 3.0	23.1 ± 3.2	0.454^•^
Smoking History			0.387^••^
Yes	15 (15.0)	20 (19.6)	
No	85 (85.0)	82 (80.4)	
Alcoholic History			0.238^••^
Yes	16 (16.0)	23 (22.5)	
No	84 (84.0)	79 (77.5)	
Snoring History			0.259^••^
Yes	12 (12.0)	18 (17.6)	
No	88 (88.0)	84 (82.4)	
ASA class			0.427^••^
1	11 (11.0)	13 (12.7)	
2	45 (45.0)	35 (34.3)	
3	40 (40.0)	47 (46.1)	
4	4 (4.0)	7 (6.9)	
Reason for ERCP			0.840^••^
Bile duct cancer	21 (21.0)	25 (24.5)	
Pancreatic cancer	15 (15.5)	12 (11.8)	
Other malignancy	8 (8.0)	7 (6.9)	
Choledocholithiasis	44 (44.0)	49 (48.0)	
Miscellaneous	12 (12.0)	9 (8.8)	
Type of ERCP			0.63^••^
Diagnostic	13 (13.0)	9 (8.8)	
Diagnostic and therapeutic	81 (81.0)	87 (85.3)	
Failed cannulation	6 (6.0)	6 (5.9)	

^•^Analyzed with the independent two sample t-test,

^••^Analyzed with the Chi-square or Fisher’s exact test

### Drug requirements and procedure duration

Patients in the Combination Group required significantly less propofol compared to the Conventional Group (135.0 ± 68.8 mg vs. 165.3 ± 81.7 mg, P = 0.005). Procedure duration of the Conventional Group and the Combination Group were not significantly different (19.9 ± 13.4 and 21.4 ± 13.6 minutes, respectively, P = 0.445) (Table 2).

**Table 2:** Drug requirements and procedure duration. Values are mean ± SD (range).

	Conventional Group (n = 100)	Combination Group (n = 102)	P-value^•^
Drug requirements			
Propofol (mg)	165.3 ± 81.7 (40 to 390)	135.0 ± 68.8 (30 to 360)	0.005
Fentanyl (μg)	-	107.2 ± 46.8 (40 to 300)	-
Propofol (μg/kg/min)	185.5 ± 134.5	129.6 ± 77.5	< 0.001
Procedure duration (min)	19.9 ± 13.4	21.4 ± 13.6	0.445

^•^Analyzed with the independent two sample t-test

### Cardiovascular and respiratory parameters

Cardiovascular and respiratory data were shown in Table 3. Baseline vital signs including mean blood pressure, heart rate and SpO_2_ were not different between the two groups. The number of patients that presented with significant hypertension or hypotension was not different between the two groups. Similarly, there was no difference in the incidence of tachycardia or bradycardia between groups. Two patients (2.0%) in the Conventional Group and 3 patients (2.9%) in the Combination Group experienced desaturation (P = 0.287), and all events were resolved with a temporary increase in oxygen flow without the need of scope removal. None of the patients required vasopressors, endotracheal intubation or premature termination of ERCP due to sedation-related complications.

**Table 3:** Cardiovascular and respiratory parameters. Values are mean ± SD or n (%) of patients. MBP, mean blood pressure. HR, heart rate

	Conventional Group (n = 100)	Combination Group (n = 102)	P-value
Baseline MBP (mmHg)	87 ± 15	90 ± 16	0.160^•^
Hypertension	22 (22.0)	21 (20.6)	0.806^••^
Hypotension	9 (9.0)	11 (10.8)	0.671^••^
Baseline HR (beats per minute)	89 ± 13	91 ± 19	0.283^•^
Tachycardia	34 (34.0)	39 (38.2)	0.531^••^
Bradycardia	4 (4.0)	3 (2.9)	0.681^••^
Baseline SpO_2_ (%)	99.4 ± 1.6	99.6 ± 0.9	0.237^•^
SpO_2_ < 90%	2 (2.0)	3 (2.9)	0.287^••^

^•^Analyzed with the independent two sample t-test,

^••^Analyzed with the Chi-square or Fisher’s exact test

### Recovery profiles and satisfaction scores

Modified Aldrete scores at 0, 5, 15 and 25 minutes after the procedure were not different between the two groups at any of the time points. Recovery times of the Conventional Group and the Combination Group were also comparable between groups [13 (5, 20) and 12 (5, 19) minutes, respectively, P = 0.662] (Table 4). Satisfaction scores of the endoscopists and patients were also not different between the two groups (P = 0.868 and 0.890, respectively) (Table 5).

**Table 4:** Recovery profiles. Values are median (IQR)

	Conventional Group (n = 100)	Combination Group (n = 102)	P-value^•^
Aldrete score at 0 min	8 (7, 9)	8 (7, 9)	0.217
Aldrete score at 5 min	9 (8, 10)	9 (8, 10)	0.379
Aldrete score at 15 min	10 (9, 10)	10 (9, 10)	0.159
Aldrete score at 25 min	10 (10, 10)	10 (10, 10)	-
Recovery time (minutes)	13 (5, 20)	12 (5, 19)	0.662

^•^ Analyzed with the Mann-Whitney U Test

**Table 5:** Satisfaction scores of the endoscopist and patients. Values are median (IQR)

	Conventional Group (n = 100)	Combination Group (n = 102)	P-value^•^
Endoscopist	90 (80, 100)	90 (80, 100)	0.868
Patient	90 (90, 100)	90 (80, 100)	0.890

^•^ Analyzed with the Mann-Whitney U Test

### Pain scores and rescue drug administration

Pain scores were significantly lower in the Combination Group compared to the Conventional Group at post-procedure 6 hours (2.5 ± 2.5 vs. 3.2 ± 2.4, P = 0.045) but not at 12, 18 or 24 hours. The frequency of rescue analgesics administration was also significantly lower in the Combination group during the first 12 hours after the procedure (0.08 ± 0.9 vs. 0.5 ± 0.8, P = 0.023) but not between 12 and 24 hours. There was no difference between the two groups in the number of times rescue anti-emetics were given during 24 hours after the procedure (Table 6).

**Table 6:** Pain scores and frequency of rescue drug administration. Values are mean ± SD. VAS, visual analogue scale

	Conventional Group (n = 100)	Combination Group (n = 102)	P-value^•^
Pain score (VAS 0~10)			
Post-procedure 6hr	3.2 ± 2.4	2.5 ± 2.5	0.045
Post-procedure 12hr	1.4 ± 2.1	1.6 ± 2.1	0.615
Post-procedure 18hr	1.0 ± 1.6	1.0 ± 1.7	0.898
Post-procedure 24hr	0.9 ± 1.6	0.7 ± 1.4	0.332
Rescue analgesics (n)			
Post-procedure 0–12 hrs	0.8 ± 0.9	0.5 ± 0.8	0.023
Post-procedure 12–24 hrs	0.2 ± 0.6	0.2 ± 0.4	0.247
Rescue anti-emetics (n)			
Post-procedure 0–12 hrs	0.1 ± 0.5	0.0 ± 0.2	0.07
Post-procedure 12–24 hrs	0.0 ± 0.2	0.0 ± 0.2	0.72

^•^Analyzed with the independent two sample t-test

## Discussion

Since BPS was first described by Cohen et al.,[13] propofol-based sedation methods for endoscopic procedures have gained much popularity. However, the debate on which regimen should be used is yet to be resolved. Sedation regimens cannot be standardized, but should be tailored according to the characteristics of the procedure such as the target site, duration, the general condition of the patient and nature of the intended procedure. Therefore, the relatively complex nature of ERCP procedures render it even more difficult to choose a safe and effective sedation regimen compared to simpler endoscopic procedures such as colonoscopy or esophagogastroduodenoscopy. We found in the present study that combining repeated doses of fentanyl for adequate pain control with continuous propofol infusion does not seem to prolong recovery times compared to a single dose of meperidine followed by continuous propofol infusion. Moreover, the proportion of endocopists and patients that felt ‘very unsatisfied’ with the overall sedation method were clearly smaller in the Combination Group.

Appropriate analgesia has emerged as an important aspect to consider when deciding on a sedation regime. Jeurnink et al.[10] reported that more discomfort was experienced during and immediately after the procedure in patients that underwent therapeutic ERCP compared to diagnostic ERCP. They also concluded that one-third to one-half of patients undergoing ERCP under conscious sedation experience discomfort and pain, implying that pain was often under-treated during and after these procedures. In the same vein, we found in a recent retrospective study that more opioids were administered during therapeutic ERCP compared to diagnostic procedures when performed under conscious sedation with propofol infusion.[11] The results of these studies point in the same direction—a need for more attention towards analgesia during ERCP, and especially during therapeutic procedures.

The concept of BPS basically involves a single induction dose of opioid and/or benzodiazepine, followed by incremental doses of propofol in order to achieve a targeted level of moderate sedation.[14] However, it is important to recognize that propofol does not have any inherent analgesic properties and therefore requires an adjuvant drug that will complement this aspect. The main problem with BPS thus seems to be the ‘single induction dose of opioid’ that is used at the beginning of the procedure in a form of premedication. This overlooks the fact that pain levels and procedure times differ significantly between ERCP procedures, and may be one of the factors leading to oversedation due to excessive doses of propofol. By taking this aspect into consideration, the sedation regimen of the Combination Group of the present study incorporated the repeated administration of an opioid according to the pain or discomfort of the patient during ERCP. Although meperidine has been most commonly used by many gastroenterologists during endoscopy for a long time,[9] its toxic potential makes it inappropriate for repeated administrations, and doses as low as 46 mg have been reported to produce central nervous system symptoms.[15] The active metabolite, normeperidine, has a half-life ranging between 14 to 21 hours, and its accumulation may precipitate anxiety, hyperreflexia, myoclonus, seizures and mood changes.[8] In order to avoid such side effects, fentanyl was used as the opioid in the Combination Group. Fentanyl has a more rapid onset and clearance, lacks dangerous metabolites or allergenic properties and is therefore commonly recommended as the safe alternative to meperidine for procedural sedation.[9,16]

We found in a recent retrospective analysis that providing analgesia by combining repeated doses of fentanyl with propofol could effectively reduce the risk of respiratory events when compared with propofol monosedation.[11] This result was attributed to the decreased risk of oversedation by reducing the overall dosage of propofol. However, it could not be concluded that repeated doses of fentanyl would not affect recovery profiles. We found in the present study that the fentanyl combination method did not prolong recovery times or worsen recovery profiles compared to conventional BPS. In other words, repeated administrations of fentanyl did not seem to affect patient recovery when used appropriately for procedural pain control. In addition, considering the suggested doses of propofol for sedation and maintenance of general anesthesia (25–75 μg/kg/min and 50–150 μg/kg/min, respectively),[17] the difference in propofol requirements of roughly 55 μg/kg/min between the two groups seem to have clinical significance.

The present study is notable in that patients were enrolled regardless of ASA class. While most previous studies done on sedation protocols enrolled patients of only ASA class 1–3,[18–21] we also included patients of ASA class 4. However, the incidence of desaturation was very low in both groups, and all events were corrected with a temporary increase in oxygen supply without the need to interrupt the procedure.

In terms of post-procedural pain, the Conventional Group showed significantly lower pain scores compared to the Combination Group at 6 hours after the procedure, and required less rescue analgesics during the first 12 hours. However, these results should be interpreted carefully. The difference in mean VAS scores between the two groups was only 0.7 (3.2 ± 2.4 vs. 2.5 ± 2.5), which may have questionable significance in actual clinical practice. This also applies to the results of rescue analgesics administration during the first 12 hours, which is statistically significant, but does not seem to have much clinical significance (0.8 ± 0.9 vs. 0.5 ± 0.8). Whether the combination method for sedation during ERCP allows lesser post-procedural pain remains unclear in this study.

This study has several limitations. First, this study was conducted in a single institution and enrolled only Asian patients that showed a tendency of a relatively low body mass index (22.9 ± 3.1 kg/m^2^) compared to the average Caucasian. A study including a patient population of diverse ethnicity should provide more insight with regard to drug dosage and adverse events. Secondly, we were not able to detect a difference in respiratory events between the two groups despite the significant reduction in propofol dosage in the Combination Group. Moreover, the incidence of respiratory events observed in the present study was under 3% in both groups, which is much lower than the previously reported 6% in patients that received ERCP procedures under propofol monosedation in a retrospective study.[11] Lastly, patients of the Combination Group were somewhat older that those of the Conventional Group with a mean age difference of 5 years. However, to what extent this difference may have affected the drug requirements and other outcomes are not clear. Overall, these results may imply that the most important factor in performing safe, yet effective sedation for therapeutic endoscopic procedures is not the sedative regimen, but the meticulous and careful monitoring of the sedation provider.

In conclusion, combining repeated doses of fentanyl for adequate pain control with continuous propofol infusion does not seem to prolong recovery times compared to a single dose of meperidine followed by continuous propofol infusion. The resulting decrease in overall propofol dose may have more significance in patients with poor general conditions which are not uncommon with ERCP. Considering the pharmacologic characteristics of the two opioids and the trend of higher satisfaction levels of the endoscopists and patients, combination sedation using repeated administrations of fentanyl with propofol infusion may be a safer and more effective sedation method compared to conventional BPS with meperidine during ERCP.

## Supporting Information

S1 CONSORT Checklist CONSORT 2010 checklist for reporting a randomiszed trial. (DOC)

S1 Protocol Clinical research protocol in original language (Korean). (DOCX)

S2 Protocol Clinical research protocol in English. (DOCX)

S1 Case Report Form (DOCX)

S1 Informed Consent Form (DOCX)

S1 Dataset Clinical research data of all patients. (XLSX)
