# Supplementary material for: Conventional versus Analgesia-Oriented Combination Sedation on Recovery Profiles and Satisfaction after ERCP: A Randomized Trial
Source: PLoS One. 2015 Sep 24;10(9):e0138422. doi: 10.1371/journal.pone.0138422 (PMC4581832; doi:10.1371/journal.pone.0138422)
Supplement: S1 Protocol — (DOCX) [file pone.0138422.s002.docx]

**연구 제목**

**국문**

ERCP 진정법에 있어서 통증조절에 초점을 둔 Fentanyl/propofol 복합투여와 기존의 Meperidine/propofol 진정법의 유용성, 안정성에 대한 무작위 대조 연구

**영문**

Randomized controlled study for analyzing clinical benefit of pain focused sedation with combination of Fentanyl and propofol during ERCP

**연구 목적**

ERCP (내시경적 역행성 담췌관 조영술) 시행시 진정법에 관하여 통증조절에 초점을 둔 Fentanyl/propofol 복합투여와 Meperidine/propofol 단독투여를 중심으로 한 기존의 진정법에 대해 안정성과 진정 만족도를 비교 분석하고자 한다.

**연구의 필요성 및 개요**

ERCP 를 시행함에 있어 진정을 시행하는 것은 환자의 불편감을 줄이고, 시술의 성공율을 높이기 위해 필수적이다 ([1](#_ENREF_1)). 대표적으로 propofol 이 짧은 반감기와 빠른 회복시간을 이유로 ERCP 의 진정에 있어 사용되고 있으며, 따라서 현재의 ERCP 진정법으로 시술 전 진통효과를 위해 투여하는 meperidine 과 시술시작과 시술 중 propofol 을 사용하는 meperidine/propofol 복합요법이 널리 사용되고 있다. 그러나 기존의 propofol 을 단독으로 사용하는 진정법은 빠른 진정효과는 있으나, therapeutic window 가 좁아 과진정, 무호흡, 산소포화도 저하와 같은 문제를 일으킬 수 있으며, meperidine 을 시술 전 한차례만 투여하므로 진통효과가 충분치 않고, 이에 따라 propofol 의 요구량이 늘어나는 한계가 있다. 이에 본원에서는 기존의 고용량 propofol 투여의 단점을 보완하고자 진통효과가 있는 Fentanyl 을 복합 투여하여 propofol 의 필요용량을 줄이고자 하였으며, 이를 통해 기존의 진정법에 비해 진정의 질 (안정성, 환자와 내시경 시술 의사의 만족도) 이 얼마나 높아지는 지를 전향적으로 무작위 비교대조분석 하고자 한다.

**피험자수 산출근거**

기존의 ERCP 진정법에 대해 분석한 전향적 무작위 비교연구를 참고하면([2](#_ENREF_2)) 제 1 종 오류 5% 그리고 통계적 검정력(power) 85%로 하여 (α=0.05, 1-β(power)=0.85) 20% 의 recovery time 을 통계학적으로 의미 있게 도출하기 위해 각 군에 각각 100명씩 연구대상으로 포함시킨다면 통계적으로 유의성을 얻을 수 있을 것으로 생각되어 총 200 여명의 환자가 필요한 것으로 산출된다.

**선정기준**

1. ERCP 를 시행하는 환자

2. 만 20세 이상의 성인

**제외기준**

1. 계란, 콩, 아황산염 포함된 방부제에 알레르기가 있는 자

2. ASA class V 환자

3. 임산부나 수유중인 자

4. 기저 질환 및 혈역학적 불안정으로 ERCP시행이 어렵다고 판단되는 자

5. 임상시험에 동의하지 않는 자

**연구 수행장소 및 기간**

연구 수행장소 : 서울특별시 서대문구 신촌동 134 세브란스병원

기간 : IRB 승인일~1년간 (200명의 환자 등록까지)

**스크리닝 방법**

1. 키, 몸무게, 흡연력, 음주력, snoring history, 최근 2주이내 URI Sx

2. 최초내원일, 주증상, 증상기간, ECOG 수행능력 (ECOG-PS)

3. 활력징후 (혈압, 맥박, 체온, 호흡수)

4. 신체검사

- General appearance, Skin, Ears-Nose-Throat, Eyes, Cardiovascular system, Breasts, Chest/Lungs, Abdomen, Genitalia, Musculoskeletal, Neurological status, Lymph nodes, Rectal, Pelvic, Other specify 에 대해 정상, 이상 여부를 판정하고 이상이 있는 경우 가능한 구체적으로 소견을 기록한다.

5. 과거력 및 진단 시기

6. 암 가족력

**대상질환의 표준 진단 및 치료 방법**

ERCP 진정법에 있어서 propofol 을 기반으로 한 진정법이 대표적으로 사용되고 있으며, 단독 투여하거나, opioid 를 복합하여 투여하는 것이 대표적 진정법으로 행해지고 있다. 그러나 대표적으로 명확하게 정해진 ERCP 진정 프로토콜은 없는 실정이다.

**연구 방법**

본 연구는 연세대학교 세브란스 병원에 내원하여 ERCP를 시행하는 환자를 대상으로 한다. 연구 기간은 IRB 승인일로부터 1년간으로 설정하여 무작위로 두 군으로 분류하여 실험군과 대조군에 각각 100명씩 200명의 환자수를 모집하는 것을 목표로 한다. 컴퓨터 기반 난수표를 기반으로 하여 양 군에 무작위 배정이 되며, 난수표가 들어있는 봉투는 본 연구에 등록된 환자가 ERCP 시행을 위해 ERCP 검사실로 이동할 때 함께 전달되어 환자가 ERCP 방에 도착하면 진정을 시행하는 마취과 의사에게만 공개되어 각 군의 프로토콜에 맞추어 검사를 진행하게 된다. 1년간 목표 환자 수에 도달하지 못할 경우 연구기간을 늘리는 것을 고려한다. 환자의 진정도는 두군 모두에서 MOAA/S (Modified Observer’s Assessment of Alertness/Sedation Scale) 3~4점을 유지하는 것을 목표로 한다. 구체적인 진정방법은 실험군의 경우에는 진정 유도시 fentanyl (Hana pharmaceutical, Korea) 1ug/kg 의 정맥주사 와 propofol (Dongkook pharmaceutical, Korea) 0.4mg/kg 의 정맥주사를 투여하며, propofol 30ug/min/kg 를 automated pump (Terufusion syringe pump TE-331, Terumo Corporation, Tokyo, Japan) 를 이용하여 정맥투여로 유지한다. 이후 추가적인 통증 조절이 필요할 시에는 fentanyl 0.5ug/kg 을 정맥투여하며, 추가적인 진정 필요 시 propofol 10mg 을 추가로 정맥투여하고 baseline propofol infusion rate를 10ug/min/kg 증량한다. 대조군의 경우에는 시술 시작 직전 meperidine 25mg (Jeil pharmaceutical, Korea) 을 정맥주사하고, 진정 유도시 propofol 1mg/kg 를 정맥투여하며, 60ug/min/kg 로 정맥투여를 유지한다. 추가적인 진정 필요시 propofol 10mg 을 추가 정맥투여하고 baseline propofol infusion rate를 10ug/min/kg 증량한다. 연구에 환자가 등록되었을 경우 내시경을 시행하는 의사와 환자 모두 어떤 군에 속하는지 알지 못하는 상태에서 진행을 하게 되며, 진정 전 환자의 생체 징후 (혈압, 맥박, 산소포화도) 를 측정하고, 진정 시작하는 시점부터 내시경 시술이 끝날 때까지 5분 간격으로 내시경실 내의 숙련된 간호사가 내시경 전문의의 지도 하에 생체 징후를 측정하고, 환자 진정 중 무호흡 (환자의 자발적 호흡이 30초 이상 억제된 경우), 저산소증 (산소포화도가 90% 미만으로 감소하여 마취과 의사의 판단 하에 jaw thrust를 시행해야 하는 경우) 발생하거나, 저산소증 이나 무호흡으로 시술을 중단하고, Ambu bagging을 시행해야 하는 경우의 발생시 추가기재 한다. 시술이 끝난 후에는 진정을 위해 환자에게 투여된 약물 (Fentanyl, propofol) 의 총량을 기입한다. 시술 직후부터 환자의 의식이 완전히 회복될 때까지는 (Modified Aldrete score = 10) 회복실의 간호사가 첫 15분 동안에는 지속적인 모니터링을 하며, 이후에는 15분 간격으로 생체 징후, 의식 회복 정도를 측정하도록 한다. ERCP 시술이 끝나고 환자의 의식이 완전히 회복되면 환자진정법의 안정성, 내시경 시술 의사와 환자의 진정에 대한 만족도 (VAS (0~100), 0 = least satisfied, 100 = most satisfied) 를 평가한다.

**안전점검자(연구자)에 의한 자료안전모니터링계획:**

이 연구는 ERCP 의 진정법으로 가장 널리 사용되는 propofol 을 기반으로 한 연구로 그 위험도가 최소 수준이다. PI 혹은 PI의 위임을 받은 연구자 (오탁근 등)가 PI의 관리감독하에 모니터링을 시행하고자 함. 첫 례와 매 50례마다 근거문서와 CRF, 프로토콜 등을 대조하여 자료의 완전성을 보증하고, 피험자의 안전성 자료를 검토하겠음.

**유효성 평가 항목**

1. Primary end point
   1. Recovery time (time from scope withdrawal to full recovery)
   2. Adverse event during and after procedure
      1. Desaturation (SaO2 < 90% for > 10 sec, need for jaw thrust) rate
      2. Apnea (cessation of respiratory activity for > 30 sec under visual observation)
2. Secondary end point
   1. Overall satisfaction with sedation and procedure by endoscopist (VAS, 0 ~ 100)
   2. Overall satisfaction with sedation by patients (VAS, 0 ~ 100)

**통계분석방법**

two sample t-test, Wilcoxon sum rank test 방법을 이용하여 연속형 자료를 분석하며, X2 square test, Fischer’s exact test 방법을 이용하여 범주형 자료를 비교 분석하고자 한다. p 값이 0.05 이하 일 때 통계적 의미가 있다고 정의한다.

**참고문헌**

1. Lee TH, Lee CK, Park SH, Lee SH, Chung IK, Choi HJ, et al. Balanced propofol sedation versus propofol monosedation in therapeutic pancreaticobiliary endoscopic procedures. Dig Dis Sci. 2012 Aug;57(8):2113-21.

2. Angsuwatcharakon P, Rerknimitr R, Ridtitid W, Kongkam P, Poonyathawon S, Ponauthai Y, et al. Cocktail sedation containing propofol versus conventional sedation for ERCP: a prospective, randomized controlled study. BMC Anesthesiol. 2012;12:20.
