# Supplementary material for: Conventional versus Analgesia-Oriented Combination Sedation on Recovery Profiles and Satisfaction after ERCP: A Randomized Trial
Source: PLoS One. 2015 Sep 24;10(9):e0138422. doi: 10.1371/journal.pone.0138422 (PMC4581832; doi:10.1371/journal.pone.0138422)
Supplement: S2 Protocol — (DOCX) [file pone.0138422.s003.docx]

**Clinical Research Protocol**

**Title:** Randomized controlled study for analyzing clinical benefit of pain focused sedation with combination of fentanyl and propofol during ERCP

**Study aim**

The present study aims to evaluate the difference in clinical outcome and satisfaction between conventional sedation with meperidine/propofol and pain focused sedation with fentanyl/propofol for ERCP.

**Background**

Adequate sedation is an essential factor of ERCP in order to reduce patient discomfort and enhance performance of the endoscopist. (1) Propofol is widely used for sedation during ERCP due to its fast onset, short half-life and rapid recovery. One of the most commonly used regimen for sedation is meperidine/propofol, which gives meperidine before the procedure for analgesia and uses propofol for sedation during the procedure. However, using propofol alone during the procedure has the downfall of possible oversedation, apnea and desaturation due to its narrow therapeutic window. Moreover, a single dose of meperidine before the procedure is often insufficient for adequate analgesia during the whole procedure, leading to an increase in propofol dose in order to overcome this problem with deeper sedation. In an attempt to supplement the lack of analgesic properties of propofol, we added fentanyl to propofol infusion during sedation for ERCP, and aim to compare this regimen with the conventional method with regard to quality of sedation and clinical outcomes in the present study.

**Sample size calculation**

Based on a previous study that compared sedation methods for ERCP (2), 100 patients are needed in each group in order to detect a 20% difference in recovery time between the two groups at a significance level of 0.05 and 85% power (α=0.05, 1-β(power)=0.85) considering a type I error of 0.05.

**Inclusion criteria**

Adult patients older than 19 years scheduled for ERCP under sedation

**Exclusion criteria**

1. Patients with known allergies to eggs, soybeans of sulfites

2. ASA class V

3. Pregnant of breastfeeding patients

4. Patients with hemodynamic instabilities that are considered unfit for ERCP under sedation.

5. Patient refusal and those unable to provide informed consent

**Institution and study period**

Institution: Yonsei University College of Medicine, Severance Hospital

Study period: 12 months following approval of IRB

**Screening**

1. Height, weight, history of smoking and alcohol intake, history of snoring, any symptoms of upper respiratory infection within 2 weeks

2. Initial visit, primary symptoms, duration of symptoms, ECOG performance status

3. Vital signs (blood pressure, pulse rate, temperature, respiratory rate)

4. Physical examination: Determine status of general appearance, skin, ears-nose-throat, eyes, cardiovascular system, chest/lungs, abdomen, musculoskeletal, neurological status

5. Underlying diseases

6. Family history of malignancies

**Standard diagnosis and therapy for studied disease**

Propofol based sedation with or without supplementary opioids is the most commonly used method for sedation during ERCP. However, there is no protocol that is accepted as standard sedation.

**Study design and method**

This study will be conducted on patients scheduled for elective ERCP at Yonsei University College of Medicine, Severance Hospital. The study period is 1 year after IRB approval. Patients will be randomized to either the control of study group until 100 patients are enrolled in each group. Randomization will be carried out by using a computer generated table of random numbers. The allocation sequence is sealed in an envelope and revealed only to the attending anesthetist providing sedation when the patient arrives at the endoscopic unit. The anesthetist will perform sedation according to the allocated group, and the patients, other study investigators and medical personnel will remain blinded to sedation method. If the targeted amount of patients are not recruited within 12 months, the study period will be extended.

Targeted level of sedation is MOAA/S (Modified Observer’s Assessment of Alertness/Sedation Scale) score 3 to 4 in all patients. Sedation in the Conventional Group is done by administrating 25 mg of IV meperidine (Pethidine, Jeil Pharmaceutical Co. Lt., Daegu, Korea) and 1 mg kg^1-^ of propofol (Pofol, Dong Kook Pharmaceutical Co. Ltd., Seoul, Korea) initially, followed by propofol infusion at a maintenance rate of 60 µg min^1-^ kg^1-^ using an automated pump (Terufusion® Syringe Pump TE-331, Terumo Corporation, Tokyo, Japan). Additional doses of 10 mg of propofol will be given when patients complain of discomfort/pain, or when the endoscopist demands deeper sedation. Patients of the Combination Group will be given 1 µg kg^-1^ of IV fentanyl (Hana pharmaceutical, Korea) and 0.4 mg kg^-1^ of propofol initially, followed by propofol infusion at a maintenance rate of 30 µg min^-1^ kg^-1^. Additional doses of 10 mg of propofol will be given during insufficient sedation, while bolus doses of 0.5 µg kg^-1^ of fentanyl will be given when additional analgesia is needed as judged by the anesthetist.

Patients will be monitored for blood pressure, pulse rate and pulse oximetry every 5 minutes throughout the procedure. Any events of apnea (cessation of voluntary respiratory for more than 30 seconds), desaturation (SpO2 lower than 90% for more than 10 seconds requiring chin lift/jaw thrust), procedure interruption due to apnea and/or desaturation for ambu bagging or other airway management are noted. Total dose of sedatives and analgesics are recorded. After the procedure, the patients are moved to the sedation recovery unit and monitored for vital signs and recovery of consciousness until the Modified Aldrete Score is 10 points and the patient is fit for discharge to the general ward. When the patient is fully alert and stable, satisfaction scores are recorded in a numerical rating scale of 0 to 100 by a nurse that is blinded to sedation method. Endoscopists will give satisfaction scores at the end of procedure.

**Data safety monitoring plan**

The sedatives and analgesics that are planned to be used in this study are widely used for sedation during ERCP and considered relatively safe. Data safety monitoring will be done by the primary investigator every 50 cases by comparing reference papers, CRFs and study protocol in order to guarantee the integrity of collected data, and review safety date of enrolled patients.

**Study end point**

1. Primary end point
   1. Recovery time (time from scope withdrawal to full recovery)
   2. Adverse event during and after procedure
      1. Desaturation (SaO2 < 90% for > 10 sec, need for jaw thrust) rate
      2. Apnea (cessation of respiratory activity for > 30 sec under visual observation)
2. Secondary end point
   1. Overall satisfaction with sedation and procedure by endoscopist (VAS, 0 ~ 100)
   2. Overall satisfaction with sedation by patients (VAS, 0 ~ 100)

**Statistical analysis**

Continuous variables will be analyzed with either the independent two sample t-test or the Mann-Whitney U Test depending on distribution. Categorical variables will be analyzed by the Chi-square or Fisher’s exact test. A P value of < 0.05 is defined as statistically significant.

**References**

1. Lee TH, Lee CK, Park SH, Lee SH, Chung IK, Choi HJ, et al. Balanced propofol sedation versus propofol monosedation in therapeutic pancreaticobiliary endoscopic procedures. Dig Dis Sci. 2012 Aug;57(8):2113-21.

2. Angsuwatcharakon P, Rerknimitr R, Ridtitid W, Kongkam P, Poonyathawon S, Ponauthai Y, et al. Cocktail sedation containing propofol versus conventional sedation for ERCP: a prospective, randomized controlled study. BMC Anesthesiol. 2012;12:20.
