# Supplementary material for: Conventional versus Analgesia-Oriented Combination Sedation on Recovery Profiles and Satisfaction after ERCP: A Randomized Trial
Source: PLoS One. 2015 Sep 24;10(9):e0138422. doi: 10.1371/journal.pone.0138422 (PMC4581832; doi:10.1371/journal.pone.0138422)
Supplement: S1 Case Report Form — (DOCX) [file pone.0138422.s004.docx]

**연구일련번호**

**입원일자 [ / / ] 생년 월일:**

**Inclusion criteria**

ERCP 를 시행하는 환자

예 □ 날짜: 아니오 □

**Exclusion criteria**

1. 계란, 콩, 아황산염 포함된 방부제에 알레르기가 있는 자 예 □ 아니오 □
2. ASA class V 환자 예 □ 아니오 □
3. 임산부나 수유중인 자 예 □ 아니오 □
4. 기타 기저 질환 및 연구 시점 당시 혈역학적 불안정 등의 원인으로 ERCP 시행이 어렵다고 판단되는 자 예 □ 아니오 □
5. 임상시험에 동의하지 않는 자 예 □ 아니오 □
6. 만 20세 미만의 자 예 □ 아니오 □

상기 환자가 이번 임상 시험에 참여합니까?

예 □ CRF/환자 번호[ ] 아니오 □

**동의 일자: / / 확인 일자: / / 연구자 서명:**

**연구일련번호**

**입원일자 [ / / ]**

**환자 기록**

**환자 이름(이니셜) : 생년월일:**

성별: 남성 □ / 여성 □ 나이: [ ]세

과거력: [ ]

가족력: [ ]

음주력: [ ]

흡연력: [ ]

Snoring/최근 2주간 URI Sx 유무: [ / ]

**Reason for ERCP**

**Tumorous condition**

Bile duct cancer suspected: [□]

Pancreatic cancer suspected: [□]

Other malignancies [□]

**Miscellaneous**

Choledocholithiasis suspected [□]

Miscellaneous [□]

**Type of ERCP**

Only diagnostic [□]

Diagnostic & therapeutic [□]

Fail [□]

**ASA class**

I [□]

II [□]

III [□]

IV [□]

**연구일련번호**

**검사일자 [ / / ][년/월/일]**

**이학적 검사**

정상 □

이상 □ specifiy

**신체 검사**

Weight [ kg]

Height [ cm]

BMI [ kg/m2 ]

Temperature [ ℃]

Blood pressure [ / mm Hg]

**임상검사**

가. Hematology

Hemoglobin [ g/dL]

WBC count [ /mm3]

Platelet [ /mm3]

나. Blood chemistry

Creatinine [ mg/dL]

Total bilirubin [ mg/dL]

GGT [ IU/L]

ALP [ IU/L]

AST [ IU/L]

ALT [ IU/L]

Amylase [ IU/L]

Lipase [ IU/L]

다. 종양 표지자 검사

CEA [ ng/mL]

CA 19-9 [ U/mL]

**연구일련번호**: [ ]

**시술일자 [ / / ][년/월/일]**

**Procedure-related time**

**Induction time** (time from sedation to scope insertion) [ min sec]

**Procedure time** (time from scope intubation to scope withdrawal) [ min sec]

**Recovery time** (time from scope withdrawal to full recovery) [ min sec]

**Monitoring factor**

**During procedure**

|  | **Baseline** | **5min** | **10min** | **15min** | **20min** | **25min** | **30min** | **35min** | **40min** |
| --- | --- | --- | --- | --- | --- | --- | --- | --- | --- |
| **BP** |  |  |  |  |  |  |  |  |  |
| **HR** |  |  |  |  |  |  |  |  |  |
| **O2sat** |  |  |  |  |  |  |  |  |  |
| **Apnea** |  |  |  |  |  |  |  |  |  |
| **Desaturation** |  |  |  |  |  |  |  |  |  |
|  | **45min** | **50min** | **55min** | **60min** | **65min** | **70min** | **75min** | **80min** | **85min** |
| **BP** |  |  |  |  |  |  |  |  |  |
| **HR** |  |  |  |  |  |  |  |  |  |
| **O2sat** |  |  |  |  |  |  |  |  |  |
| **Apnea** |  |  |  |  |  |  |  |  |  |
| **Desaturation** |  |  |  |  |  |  |  |  |  |

**Procedure cessation due to desaturation or apnea** [□] ( min from sedation)

**Need for ambu bagging due to desaturation or apnea** [□] ( min from sedation)

**After procedure**

|  | **0min** | **15min** | **30min** | **45min** | **60min** |
| --- | --- | --- | --- | --- | --- |
| **BP** |  |  |  |  |  |
| **HR** |  |  |  |  |  |
| **O2sat** |  |  |  |  |  |
| **Apnea** |  |  |  |  |  |
| **Desaturation** |  |  |  |  |  |
| **Modified Aldrete Scoring** |  |  |  |  |  |

**Satisfaction score**

**Overall satisfaction with sedation and procedure by endoscopist (VAS, 0 ~ 100) [ ]**

**Overall satisfaction with sedation by patients (VAS, 0 ~ 100) [ ]**

**(0 = least satisfied, 25 = mostly dissatisfied, 50 = mixed,**

**75 = mostly satisfied, 100 = absolutely satisfied)**

**Total dose of drugs**

**Propofol [ mg]**

**Fentanyl [ ug]**

**Post-procedure pain and N/V**

|  | **6hr** | **12hr** | **18hr** | **24hr** |
| --- | --- | --- | --- | --- |
| **Pain score (VAS 0-10)** |  |  |  |  |
| **Rescue analgesics (n)** |  |  |  |  |
| **Rescue antiemetics (n)** |  |  |  |  |

**Patients’ subgroup (Blind 상태로 진행하며 모든 수치 기입 후 마지막에 기입)**

**Control group [□]**

**Experimental group [□]**

**Appendix: Experimental Protocol**

- Conventional sedation
- Meperidine : 25 mg IV just before procedure
- Propofol
- IV bolus of 1 mg/kg for sedation induction,
- Infusion of 60 µg/min/kg for maintenance of sedation
- Additional bolus dose of 10mg as needed for adequate sedation
- Experimental sedation
- Fentanyl
- IV bolus of 1 µg/kg for sedation induction
- Additional bolus dose of 0.5 µg/kg as needed for adequate pain control
- Propofol
- IV bolus of 0.4 mg/kg for sedation induction,
- Infusion of 30 µg/min/kg for maintenance of sedation
- Additional bolus dose of 10mg as needed for adequate depth of sedation
