# Supplementary material for: Conventional versus Analgesia-Oriented Combination Sedation on Recovery Profiles and Satisfaction after ERCP: A Randomized Trial
Source: PLoS One. 2015 Sep 24;10(9):e0138422. doi: 10.1371/journal.pone.0138422 (PMC4581832; doi:10.1371/journal.pone.0138422)
Supplement: S1 Informed Consent Form — (DOCX) [file pone.0138422.s005.docx]

**피 험 자 설 명 문**

| **연구제목:** | ERCP 진정법에 있어서 통증조절에 초점을 둔 Fentanyl/propofol 복합투여와 기존의 Meperidine/propofol 진정법의 유용성, 안정성에 대한 무작위 대조 연구 |
| --- | --- |
| **연구책임자:** | 연세대학교 의과대학 세브란스병원 소화기내과 교수 방승민  120-752 서울특별시 서대문구 성산로 250  ☎ 02-2228-5190 |
| **의 뢰 자:** | 방승민 |

이 설명문은 본 연구의 목적, 절차, 이익, 위험, 불편, 주의사항 등을 기술하고 있습니다. 귀하께서는 연구에 관한 다음의 설명을 읽고 충분히 이해하고 생각하신 후에 연구 참여에 동의할 지의 여부를 결정하여 주시기 바랍니다.

본 연구는 세브란스병원 연구심의위원회에서 위험 및 이익을 분석하였으며 윤리적, 과학적으로 승인된 연구입니다

**연구 수행 배경, 연구의 목적**

ERCP (내시경적 역행성 담췌관 조영술) 를 시행함에 있어 진정을 시행하는 것은 환자의 불편감을 줄이고, 시술의 성공율을 높이기 위해 필수적입니다. 대표적으로 propofol 이라는 진정제가 빠른 회복시간을 이유로 ERCP 의 진정에 사용되고 있어, 진통제인 meperidine 을 시술 전 투여하고, 시술 시에는 propofol 을 사용하는 meperidine/propofol 복합요법이 ERCP 의 진정 법으로 널리 사용되고 있습니다. 그러나 기존의 propofol 을 단독으로 사용하는 진정법은 빠른 진정효과는 있으나, 과도한 진정으로 인해 저산소증, 무호흡 등의 위험성이 있으며, 진통제인 meperidine 을 시술 전에만 한차례 투여하여 통증 경감효과가 부족할 가능성이 있습니다. 이에 본원에서는 기존의 고용량 propofol 투여의 단점을 보완하고자 진통효과가 있는 Fentanyl 을 복합 투여하여 propofol 의 투여용량을 줄이고자 하였으며, 이를 통해 기존의 진정법에 비해 진정의 질 (안정성, 환자와 내시경 시술 의사의 만족도) 이 얼마나 높아지는 지를 비교분석 해보고자 합니다.

**연구 참여 피험자 수 및 연구 기간**

이 연구는 세브란스 병원 단일 기관에서 2013년 1월부터 2013년 12월까지 200명의 피험자가 참여 등록될 예정입니다.

**시술 또는 절차에 대한 설명**

귀하께서 본 연구에 참여하시기로 결정하셨다면, 피험자 동의서 양식에 서명하시게 됩니다. 동의서에 서명하신 이후에, 연구자는 귀하의 병력 및 신체검진, 혈액검사를 포함한 검사를 시행할 것입니다.

위 검사의 결과를 통하여 귀하께서 연구 계획에 적합하다고 판단되는 경우, ERCP 를 시행하면서 두가지 방법 (기존의 진정법 또는 본 연구에서 계획하는 진정법) 중 한가지의 방법으로 진정을 진행하게 됩니다. 기존의 진정법은 진정제인 propofol 사용을 주로 하는 meperidine/propofol 복합요법이며, 본 연구에서는 연구의 목적에서 명시한 바와 같이 기존의 고용량 propofol 투여의 단점을 보완하고자 진통효과가 있는 Fentanyl 을 복합 투여하여 propofol 의 투여용량을 줄이고자 함에 있습니다.

**대체 진단 및 치료 방법**

ERCP 의 진정법으로는 propofol 을 기반으로 propofol 단독 또는 진통제가 추가되는 방법의 진정법이 널리 행해지고 있으나 명확한 진정 프로토콜은 없는 실정입니다. 본 연구에서 어떤 군에 속해도 기존에 입증된 진정법을 기반으로 진정이 진행됩니다.

**임상연구 참여에 따른 비용**

ERCP 의 진정은 필수적이며 본원에서 적용되는 두가지 진정법 모두 ERCP 의 진정으로 입증되고 널리 사용되는 약제를 사용하는 것으로써 비용은 피험자가 부담하게 됩니다. 연구에 등록됨으로써 피험자가 부담해야 할 추가 비용은 없습니다.

**상해에 대한 보상**

본 연구에 등록되지 않아도 ERCP 의 진정은 필수적이며, 사용하는 약제는 큰 변화가 없기 때문에 상해에 대한 보상은 피험자 부담이 됩니다.

**피험자가 준수하여야 하는 사항**

귀하의 안전과 정확하고 과학적인 연구에 기여하기 위해서 의료진의 지시를 따라 주시기 바랍니다.

1) 귀하는 정해진 임상시험계획에 따라 정해진 두가지 진정법 중 환자에게 적용되는 진정방법에 대해 동의하셔야 합니다.

2) 진정 후의 진정법에 대한 만족도에 대한 질문에 응해주셔야 합니다.

**연구 참여에 따른 이익**

안정성과 만족도 측면에서 질 좋은 수면진정을 기대할 수 있으며, 좀 더 체계적인 진정 전후의 평가를 받을 수 있습니다. 그러나 경우에 따라 연구에 참여함에 따른 이익이 없을 수도 있으며 금전적 보상은 없습니다.

**연구 참여에 따른 위험성 및 부작용, 불편**

각각의 진정 약제의 부작용 (과다한 진정에 따른 저산소증, 무호흡, 약물에 대한 과민반응 등) 이외에 특별한 부작용, 불편감은 없습니다.

**중도 탈락**

진정방법이 프로토콜 내에서 부득이하게 바뀌는 경우

**연구용 검체의 수집 및 보관**

본 연구를 시행함으로써 추가적인 채혈이나 조직검사, 수집, 보관이 필요하지는 않습니다.

**기록에 대한 비밀보장**

귀하께서 본 연구에 참여하시는 동안에 수집되는 귀하의 기록은 비밀로 보장될 것이며, 연구의 결과가 보고서로 작성되거나 출판, 또는 발표되는 경우에도 귀하의 신원을 파악할 수 있는 기록은 비밀 상태로 유지될 것입니다.

본 연구를 모니터/점검하는 자, 연구심의위원회(IRB) 및 식품의약품안전청장은 귀하의 비밀 보장을 침해하지 않고 관련 규정이 정하는 범위 안에서 연구의 실시 절차와 자료의 신뢰성을 검증하기 위해 귀하의 의무기록을 직접 열람할 수 있습니다. 귀하께서는 본 동의서 서식에 서명함으로써 귀하 또는 귀하의 법정대리인이 이러한 자료의 직접 열람을 허용하시게 됩니다.

**참여/철회의 자발성**

귀하께서는 임상 연구에 참여할 수도 있고 참여하지 않을 수도 있습니다. 또한 이후 언제라도 연구 참여를 그만 둘 수 있습니다.

귀하의 결정은 향후 귀하께서 받게 되실 치료에 영향을 주지 않습니다.

연구에 참여하지 않거나 중도에 그만 두기로 결정하더라도 귀하에 대한 어떠한 불이익이 발생하지 않을 것이며 귀하께서 원래 받을 수 있는 이익에 대한 어떠한 손실도 없을 것입니다.

연구에 계속해서 참여할 지의 여부를 결정하는 데에 영향을 줄 만한 새로운 정보가 수집될 경우, 연구자는 이 정보를 귀하 또는 귀하의 법정 대리인에게 적시에 알려드릴 것입니다.

**연락처**

본 연구에 관하여 궁금한 점이 있거나 연구와 관련이 있는 상해가 발생한 경우에는 아래의 연구자에게 연락하여 주십시오.

**연구자 성명 : 오탁근**

**연구자 주소 : 서울특별시 서대문구 성산로 250 세브란스병원**

**☎ 02-2228-5190 / 24시간 연락처 : 010-9263-3868**

피험자로서 귀하의 권리에 대하여 질문이 있는 경우에는 연구자에게 말씀하시거나 다음의 번호로 문의하실 수 있습니다.

**세브란스병원 연구심의위원회 ☎ 02-2228-0430~4**

**세브란스병원 임상연구보호센터 ☎ 02-2228-0450~4**

**피 험 자 동 의 서**

| **연구제목 :** | | ERCP 진정법에 있어서 통증조절에 초점을 둔 Fentanyl/propofol 복합투여의 유용성, 안정성에 대한 무작위 대조 연구 |
| --- | --- | --- |
|  |  | |
| □ | 본인은 이 설명문을 읽었으며, 본 임상연구의 목적, 방법, 기대효과, 가능한 위험성, 타 치료 방법의 유무 및 내용, 건강 정보 관리 등에 대한 충분한 설명을 듣고 이해하였습니다. | |
| □ | 모든 궁금한 사항에 대해 질문하였고, 충분한 답변을 들었습니다. | |
| □ | 본 연구에 동의한 경우라도 언제든지 철회할 수 있고, 철회 이후 다른 적절한 치료를 받을 수 있음을 확인하였습니다. | |
| □ | 본인은 설명문 및 작성된 동의서 사본 1부를 받았음을 확인합니다. | |
| □ | 충분한 시간을 갖고 생각한 결과, 본인은 이 연구에 참여하기를 자유로운 의사에 따라 동의합니다. | |

|  | | | | |
| --- | --- | --- | --- | --- |
| 연구 참여자의 성명 |  | 서명 |  | 날짜(년/월/일) |
|  | | | | |
| 법정대리인의 성명(해당되는 경우) |  | 서명 |  | 날짜(년/월/일) |
|  | | | | |
| 입회자의 성명(해당되는 경우) |  | 서명 |  | 날짜(년/월/일) |
|  | | | | |
| 설명한 연구자의 성명 |  | 서명 |  | 날짜(년/월/일) |
